# The DELIVER Technique: Deep Engagement of Guide or ChiLd-guIde Catheter for Burr DeliVEry and Rotational Atherectomy

**DOI:** 10.1016/j.jaccas.2024.103177

**Published:** 2025-03-05

**Authors:** Umihiko Kaneko, Yoshifumi Kashima, Shoichi Kuramitsu, Yutaka Tadano, Takuro Sugie, Ken Kobayashi, Daitaro Kanno, Katsuhiko Sato, Tsutomu Fujita

**Affiliations:** Department of Cardiovascular Medicine, Sapporo Cardio Vascular Clinic, Sapporo Heart Center, Sapporo, Japan

**Keywords:** calcified lesion, deep engagement, distal lesion, rotational atherectomy, tortuous vessel

## Abstract

The delivery of the rotational atherectomy burr can sometimes be hindered in distal calcified lesions complicated by proximal vessel tortuosity or other obstacles. This problem may result in procedural failure or fatal complications, including coronary perforation, burr entrapment, or driveshaft fracture. To prevent these catastrophic outcomes and ensure successful burr delivery, we introduce the DELIVER (Deep Engagement of guide catheter or 5-F chiLd-guIde catheter for burr deliVEry and subsequent Rotational atherectomy) technique. This method involves deep catheter insertion beyond proximal vessel tortuosity or other obstacles, using strategies such as the distal balloon anchoring technique. Once the catheter is positioned, the rotational atherectomy burr is advanced through it to facilitate the atherectomy of the distal target lesion. This report presents 3 cases where the DELIVER technique was applied successfully to treat distal lesions. The technique enabled smooth and atraumatic burr delivery, even through tortuous arterial segments or other challenging anatomical structures.

## Visual Summary

**Figure:**
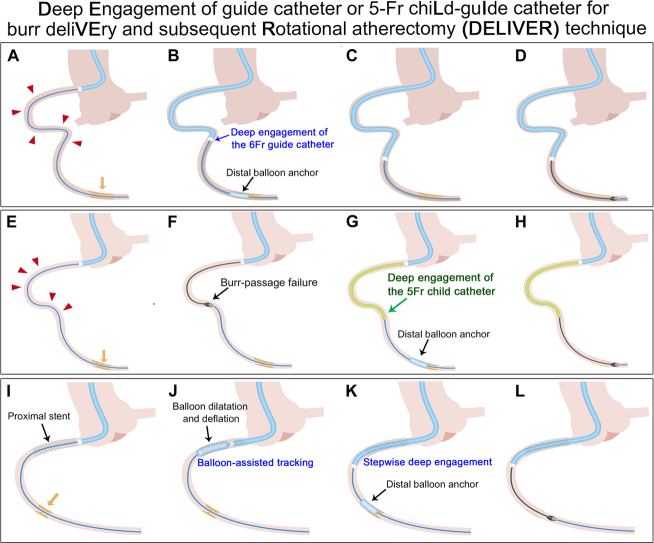

**Visual Summary:**
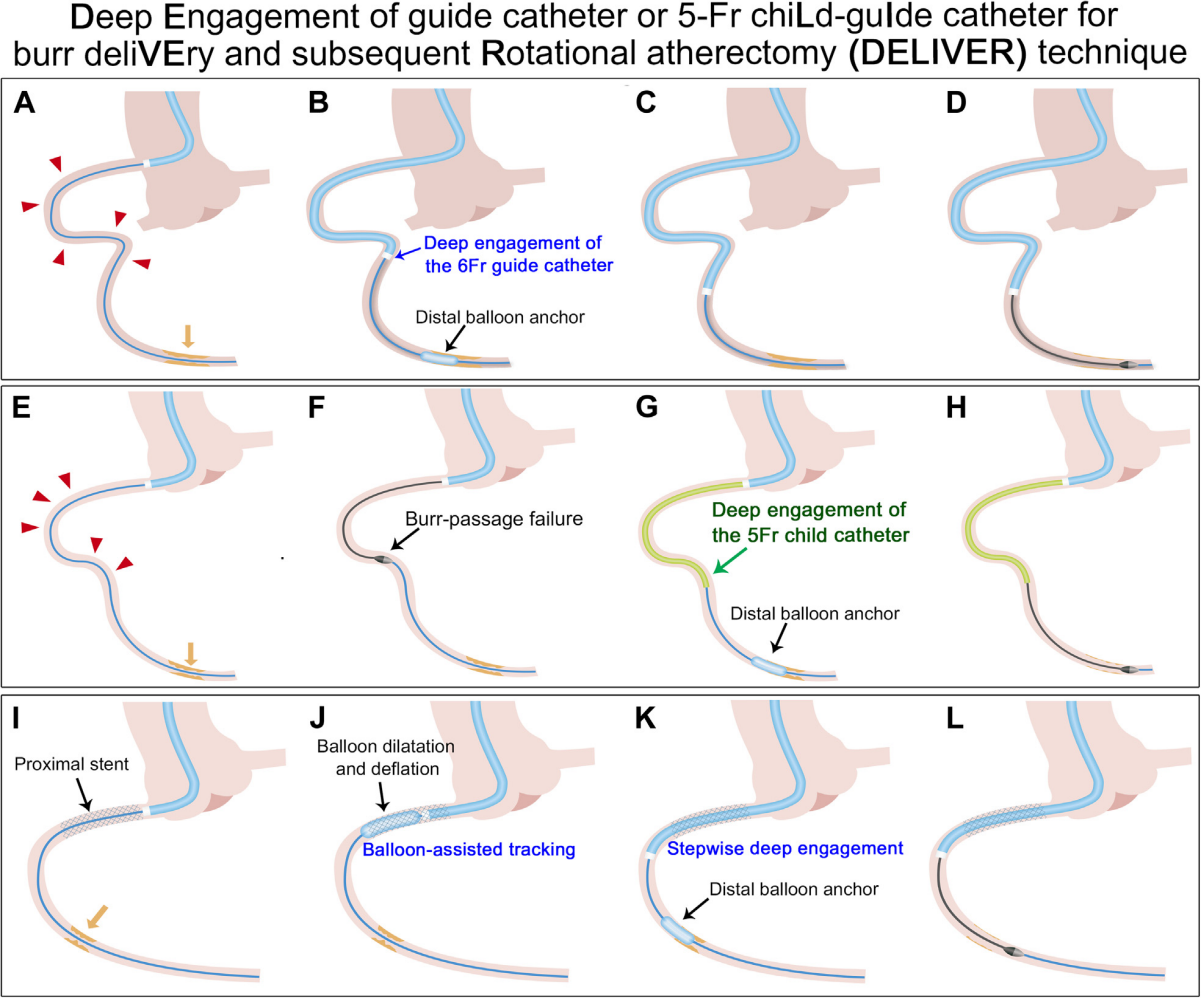
Summary of the DELIVER Technique (A) Calcified distal right coronary artery (RCA) stenosis and significant proximal vessel tortuosity. (B and C) A 6F guide catheter can be deeply inserted beyond a significantly tortuous segment via distal balloon anchoring. (D) Deeply inserted guide catheter enables safe delivery of a 1.5-mm burr distally and subsequent rotational atherectomy. (E) Baseline coronary angiogram revealing severe vessel tortuosity proximal to the calcified culprit lesion in the distal RCA. (F) Failure of distal delivery of the 1.5- or 1.25-mm burr owing to significant proximal tortuosity and calcification. (G) Deep insertion of a 5-F ST01 catheter via distal balloon anchoring. (H) Successful rotational atherectomy of the distal target lesion. (I) Baseline coronary angiography reveals a calcified mid-RCA lesion and a previously implanted stent in the ostial RCA. (J and K) Step-wise advancement of the guide catheter through the proximal stent via balloon-assisted tracking. (L) Successful delivery of a 1.75 mm burr and rotational atherectomy runs. Red arrowheads; proximal tortuous segment; yellow arrows, distal calcified lesion.

Percutaneous coronary intervention for heavily calcified lesions remains challenging because of the higher incidence rates of periprocedural complications and poor clinical outcomes.^1^^,^^2^ Rotational atherectomy (RA) is the standard debulking procedure for heavily calcified plaques, and decades of clinical evidence and experience have standardized the RA technique, resulting in a high procedural success rate.^3^, ^4^, ^5^, ^6^, ^7^

**Take-Home Messages**

• RA for distal target lesions complicated by proximal vessel tortuosity, anomalous coronary arteries, or other obstacles remains unestablished, because it is associated with a high risk of fatal proximal vessel injury or delivery failure.
• The DELIVER technique enables smooth and atraumatic burr delivery, even through tortuous arterial segments or other challenging anatomical structures.

**Figure 1:**
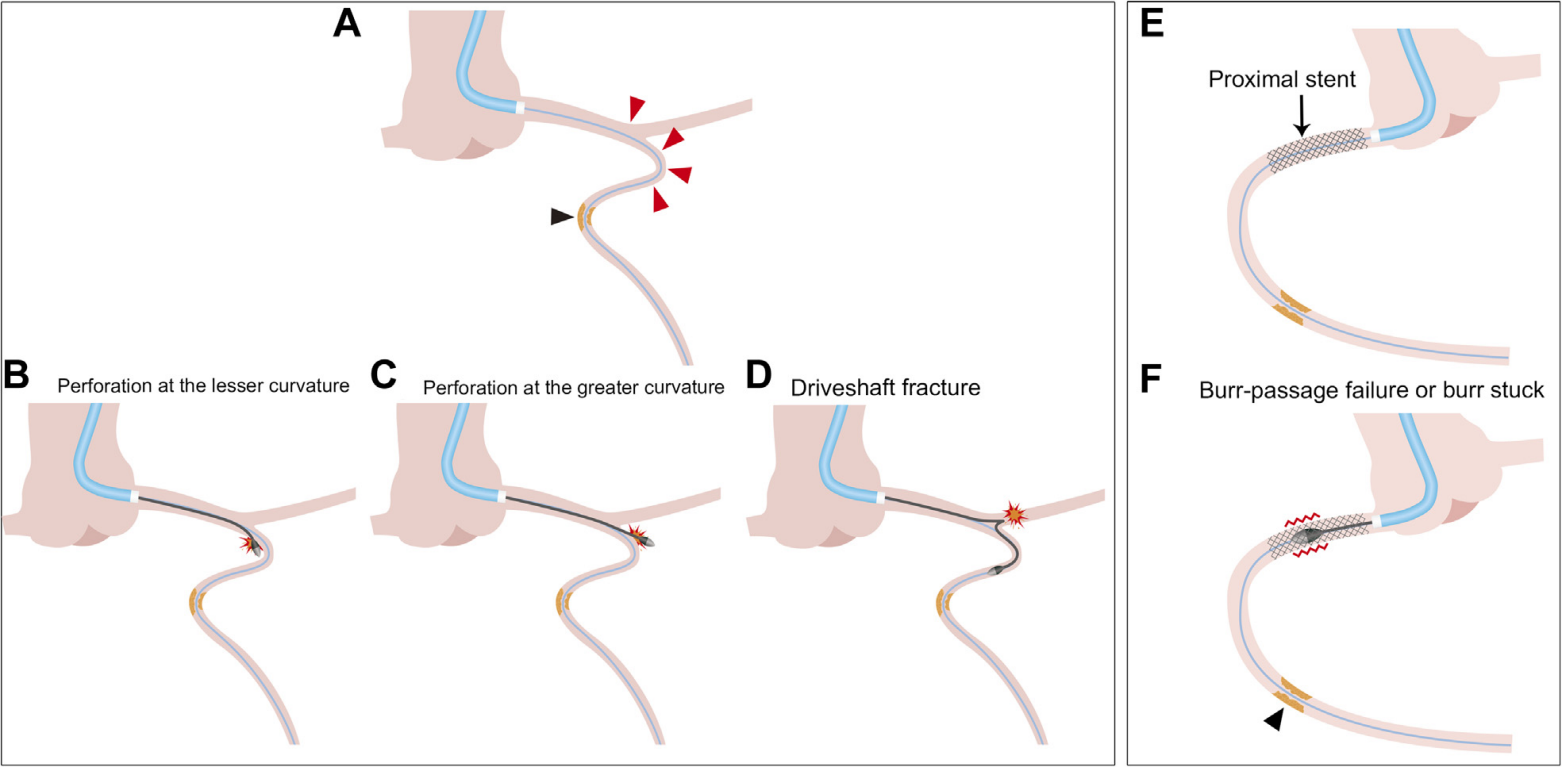
Possible Difficulties of Rotational Atherectomy for Distal Calcified Lesions in Tortuous Proximal Vessel Segments or Coronary Stents (A) A distal calcified lesion across a tortuous proximal vessel segment. (B) Deep cut at the lesser curvature. (C) Coronary perforation at the greater curvature. (D) Driveshaft fracture at the tortuous proximal vessel segment. (E) A distal calcified lesion across a proximal coronary stent. (F) Burr-passage failure or burr entrapment. Black arrowheads, distal calcified lesion; red arrowheads, proximal tortuous segment.

Nevertheless, a standardized treatment strategy for RA of distal target lesions complicated by proximal tortuosity, anomalous coronary arteries, or obstacles—such as previously implanted stents, or transcatheter aortic valve replacement valve—has yet to be established.^5^^,^^8^^,^^9^ The distal location of some lesions often impacts procedural outcomes, impairing the deliverability of the RA burr. Furthermore, performing RA on such lesions carries a high risk of burr entrapment and fatal vessel injuries, including coronary perforation, dissection, and Rotablator driveshaft fracture in the proximal vessel (Figures 1A to 1F).^8^

Here, we introduce the Deep Engagement of guide catheter (GC) or 5F chiLd-guIde catheter (5CG) for burr deliVEry and Rotational atherectomy: the DELIVER technique. This method involves deep insertion of 5CG or a standard GC beyond proximal vessel tortuosity or obstacles, using techniques such as distal balloon anchoring. Once the catheter is in position, the RA burr is advanced through it to perform RA on the distal target lesion while minimizing the risk of proximal vessel injury. We present 3 cases of successful RA for distal lesions using the DELIVER technique. The study conforms with the Declaration of Helsinki guidelines and was approved by the Ethical Committee of the Sapporo Cardio Vascular Clinic (Approval No. 20230012).

## Case 1

**Figure 2:**
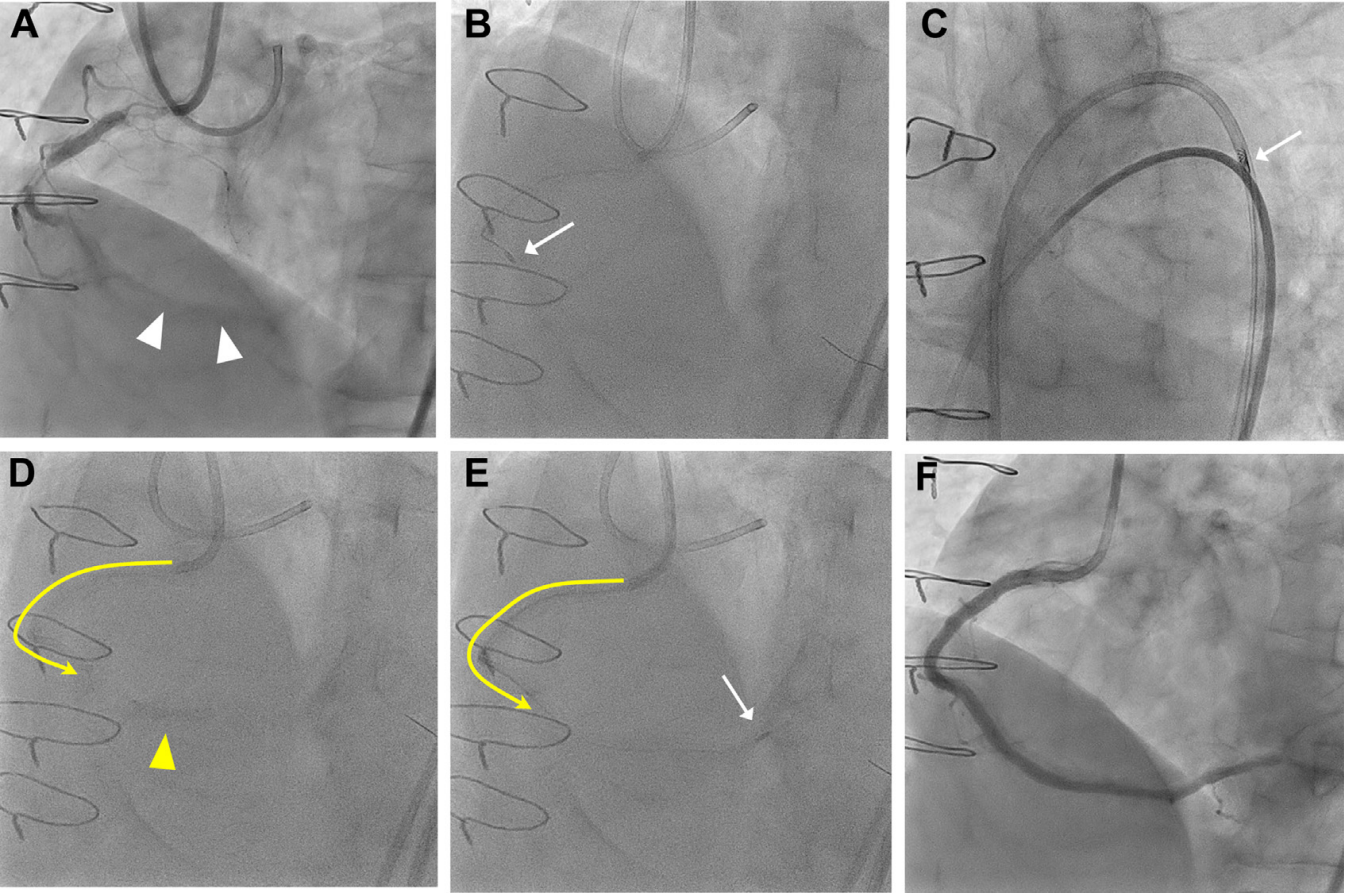
Deep Engagement of a 5F Child Guide Catheter Through an Extremely Long and Tortuous RCA and Subsequent Rotational Atherectomy (A) Baseline coronary angiography reveals the severe vessel tortuosity proximal to the occlusion and calcified culprit lesion (white arrowheads) in distal RCA. (B) Failure of the distal delivery of the 1.5- or 1.25-mm burr owing to significant proximal vessel tortuosity and calcification. (C) The 1.25-mm burr is unable to pass through a 7-F guide extension catheter at the entry port. (D) Deep insertion of the 5-F ST01 (Terumo) catheter (yellow line with arrow) via distal balloon anchoring (yellow arrowhead). (E) Smooth passage of a 1.25-mm burr (white arrow) through the 5-F ST01 to a point just proximal to the distal target lesion; subsequent rotational atherectomy is successful. (F) Final angiography demonstrates the excellent results. RCA = right coronary artery.

A 68-year-old man with a history of inferior myocardial infarction underwent percutaneous coronary intervention for occluded and calcified lesions in the proximal to distal portion of the right coronary artery (RCA) (Figure 2A, Video 1). A Mach1 8-F FCR 3.5 GC (Boston Scientific) was inserted via the right femoral artery. Although the proximal and mid calcified lesions were ablated with a 1.5-mm burr after guidewire crossing, distal delivery of the 1.5- or 1.25-mm burr failed owing to significant proximal vessel tortuosity and calcification (Figure 2B, Video 2). Although we inserted a 7-F guide extension catheter (GEC) (GUIDEZILLA Ⅱ, Boston Scientific), the 1.25-mm burr could not pass through the GEC’s entry port (Figure 2C). Next, we inserted the 5CG catheter (Heartrail ST01/5Fr, Terumo) beyond the tortuous segment of the mid-RCA via distal balloon anchoring technique (Figure 2D, Video 3). The 1.25-mm burr easily passed through the 5CG using the Dynaglide mode, and subsequent RA for the distal target lesion was successfully performed with adequate support from the 5CG (Figure 2E, Video 4). After pre-dilatation and implantation of 3 drug-eluting stents, final angiography and intravascular ultrasound revealed excellent results (Figure 2F).

### Follow-up

At the 1-year clinical follow-up, the patient was stable and asymptomatic.

## Case 2

**Figure 3:**
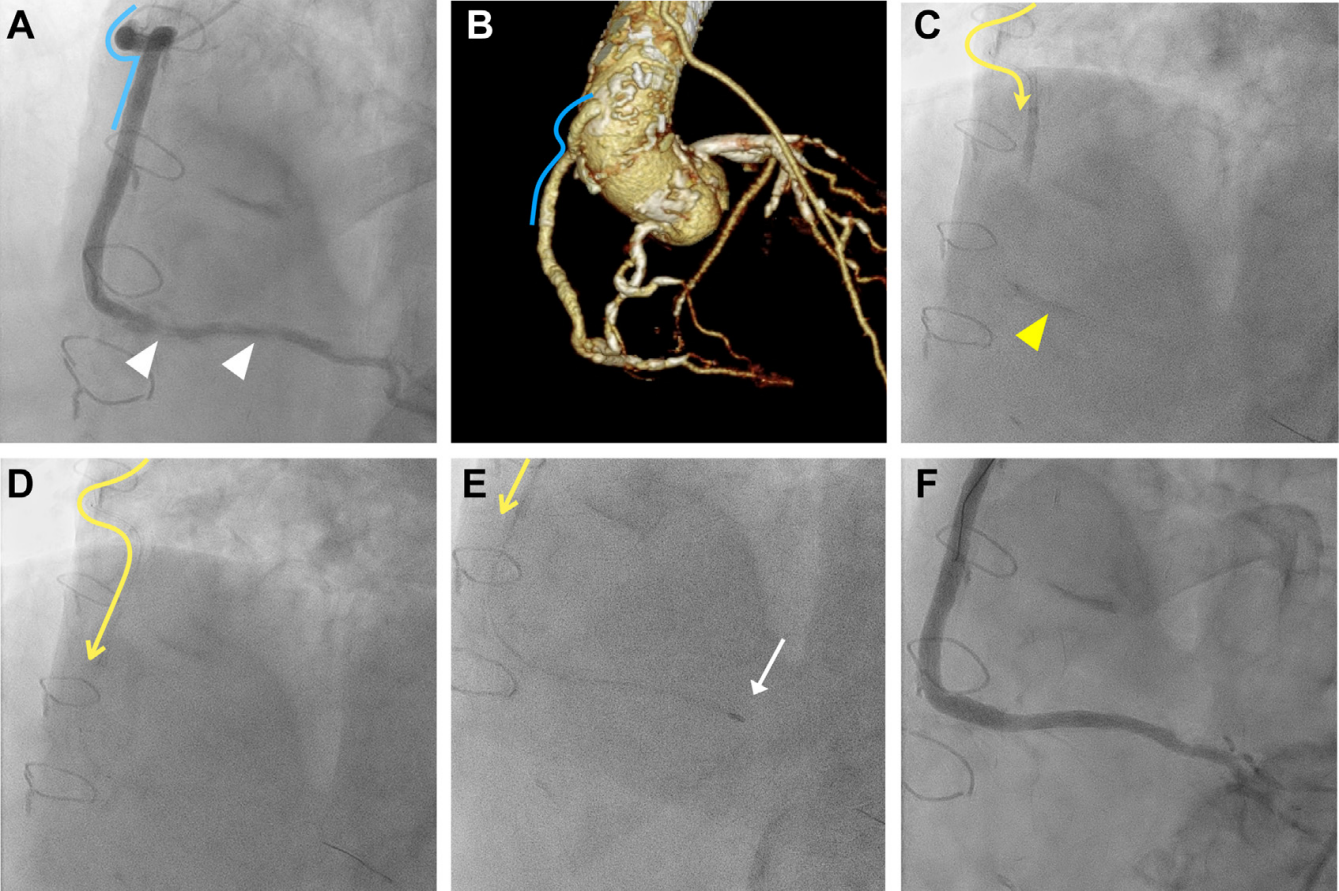
Deep Engagement of a 6-F Guide Catheter Through a Tortuous Saphenous Vein Graft and Rotational Atherectomy for Distal RCA Lesion (A and B) Baseline coronary angiography and computed tomography reveals calcified distal RCA stenosis and a significantly tortuous proximal saphenous vein graft (blue line) anastomosed to the mid-RCA. (C and D) A 6-F Judkins right 4.0 guide catheter (yellow line with arrow) is deeply inserted beyond the tortuous saphenous vein graft segment via distal balloon anchoring technique (yellow arrowhead). (E) Deeply inserted guide catheter enables safe delivery of the 1.5-mm burr (white arrow) distally and subsequent rotational atherectomy. (F) Final angiography demonstrates the excellent results. Abbreviation as in Figure 1.

An 80-year-old man with a history of coronary artery bypass graft surgery presented with a significant stenotic and heavily calcified lesion in the distal RCA, along with a chronic total occlusion in the mid-RCA (Figures 3A and 3B). Consequently, percutaneous coronary intervention was performed via the tortuous saphenous vein-RCA graft to address the heavily calcified distal RCA lesion. Owing to severe tortuosity in the proximal saphenous vein, which posed a high risk for RA, a Hyperion 6-F J Judkins Right 4.0 GC (Asahi Intec) inserted via the right femoral artery was intentionally advanced deep beyond the tortuous segment with the assistance of distal balloon anchoring (Figures 3C and 3D, Video 5). The deeply positioned GC acted as a protective sheath, facilitating the safe delivery of a 1.5-mm burr to the distal lesion, which enabled successful RA (Figure 3E, Video 6). The lesion was then revascularized with stenting and post-dilatation (Figure 3F).

### Follow-up

At the 8-month follow-up, the patient was asymptomatic and coronary computed tomography angiography showed no restenosis of the lesion.

## Case 3

**Figure 4:**
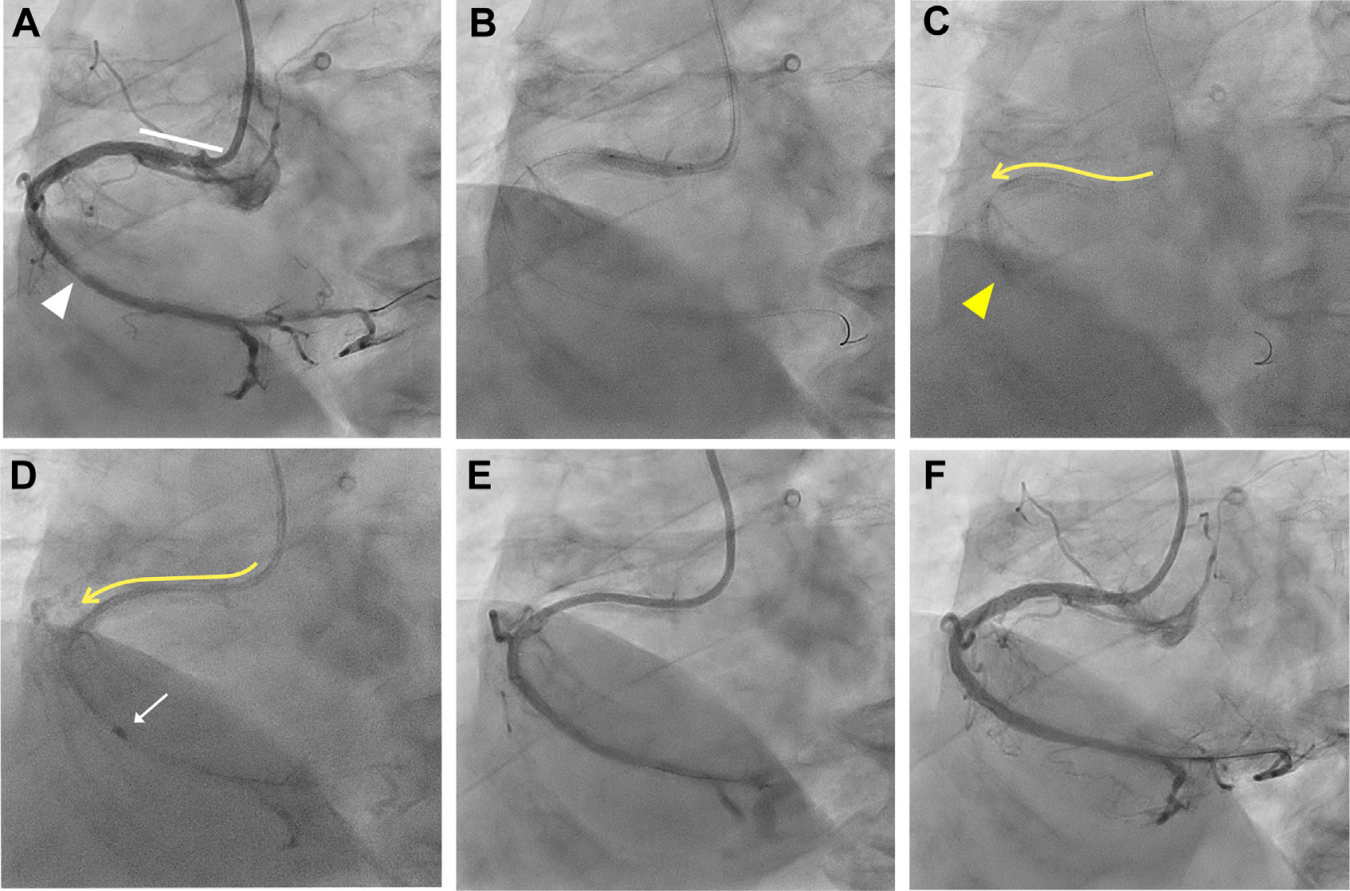
Deep Engagement of a 6-F Guide Catheter Through a Stent in ostial RCA and subsequent RA for distal RCA lesion (A) Baseline coronary angiography reveals a calcified mid-RCA lesion (white arrowhead) and previously implanted stent in ostial RCA (white line). (B) Step-wise advancement of the guide catheter through the proximal stent by repeated 3.5-mm balloon dilatation and deflation (balloon-assisted tracking). (C) Further deep insertion of the guide catheter (yellow line with arrow) aided by distal balloon anchoring (yellow arrowhead). (D) Successful delivery of a 1.75-mm burr (white arrow) and rotational atherectomy. (E) Angiography after rotational atherectomy confirms improved coronary flow and enlarged lumen. (F) Final angiography demonstrates the excellent results. RA = right atrial; other abbreviation as in Figure 1.

An 83-year-old woman with a history of stent implantation in the ostial RCA had a significant stenotic and calcified lesion in the mid-RCA (Figure 4A). Selective engagement of a 6-F Heartrail Ⅱ Ikari Left 3.5 GC, via a right transfemoral approach, was hindered by the previously implanted aorto-ostial stent. Owing to the significant friction generated by the ostial RCA stent, the 1.5-mm burr delivery to the mid-RCA was unsuccessful. Furthermore, deep advancement of the GC beyond the ostial stent using a distal balloon anchor failed because of interference between the ostial stent and the GC. Subsequently, further advancement of the GC was successfully achieved with repeated 3.5-mm noncompliant balloon dilatations and simultaneous deflation to allow stepwise advancement of the GC through the ostial stent (combination of balloon-assisted tracking and distal balloon anchor technique) (Figures 4B and 4C, Video 7).^10^ A 1.5-mm burr was smoothly advanced through the GC, enabling the successful completion of multiple RA procedures. Owing to residual calcification, additional RA was performed using a 1.75 mm burr (Figures 4D and 4E, Video 8). The lesion was revascularized after pre-dilatation and stenting (Figure 4F). The final intravascular ultrasound assessment confirmed the absence of proximal stent deformation.

### Follow-up

One year later, the patient remained stable, and coronary computed tomography angiography confirmed good patency of the lesion.

## Discussion

This report presents 3 cases of successful RA for distal lesions performed with the DELIVER technique, which allows easy and atraumatic burr delivery over tortuous artery segments or obstacles.

In the coronary artery, target vessel tortuosity increases the risk of clinical and procedural RA failure.^9^ In particular, a proximal vessel tortuosity or obstacles can limit burr deliverability to a distal target lesion, thus rendering all RA procedures much riskier and more complex, and burr delivery often fails. RA for the distal lesions involves a high risk of fatal vessel injury (coronary perforation/dissection), burr entrapment, or Rotablator driveshaft fracture in the proximal vessel (Figures 1A to 1F).^5^^,^^8^^,^^9^ As a result, the DELIVER technique is essential to address these challenging situations.

The DELIVER technique comprises the following 3 steps:

1. Intentional deep engagement of the GC or 5CG beyond the proximal vessel tortuosity or obstacles using distal balloon anchoring, balloon-assisted tracking, inchworm technique, or combinations of these techniques
2. Delivery of the RA burr to the distal target lesion through the GC or 5CG
3. RA performed supported with the deeply engaged GC or 5CG.

This technique is effective for delivering the burr: !) across the proximal vessel tortuosity; @) in anomalous coronary arteries; #) through the proximal stent; or $) through the transcatheter aortic valve replacement valve.

The DELIVER technique offers several practical advantages. 1) The catheter can be easily advanced past proximal vessel tortuosity or obstacles using one or a combination of the 3 catheter advancement strategies outlined below. 2) Both the GC and the 5CG feature smooth, tubular lumens that facilitate the easy delivery of the RA burr, whereas the GEC often impedes burr passage owing to a significant gap at its entry port. 3) The RA burr can be advanced distally through the deeply positioned catheter, bringing the platform close to the distal target lesion. 4) The deeply positioned catheter provides sufficient back-up support during ablation. 5) The catheter acts as a protective sheath, decreasing the risk of RA-related proximal vessel injury.

When actually performing RA with the DELIVER technique, plain advancement of the 5CG or GC is often compromised owing to strong resistance in the proximal vessel tortuosity or obstacles. For this reason, 3 specific catheter advancement strategies are mandatory: 1) distal balloon anchor; 2) balloon-assisted tracking; and 3) the inchworming technique.^10^, ^11^, ^12^, ^13^, ^14^ The distal balloon anchor technique—balloon inflation in the distal lesion as an anchor and simultaneously pulling-up the balloon—can help to advance the catheter over the balloon shaft through the proximal obstacles. When catheter advancement is hindered owing to interference by the proximal stent or transcatheter aortic valve replacement valve, balloon-assisted tracking or inchworming technique can help to overcome these problems. In the balloon-assisted technique, repeated balloon dilatation just distal to the catheter inside the proximal obstacle and subsequent catheter advancement during balloon deflation are possible. The inchworming technique—a small balloon (eg, 2.0 mm) is positioned at the tip of the catheter, inflated at low pressure, and the catheter is then advanced during balloon deflation. In summary, these 3 strategies, used alone or in combination, are the most efficient ways to securely advance the catheter across tortuous vessels or obstacles.

Currently, 3 types of catheters are available for deep insertion to delivery the RA burr distally: 1) 5CG; 2) GC; and 3) GEC, each with advantages and disadvantages. (Table 1) A 5CG (Heartrail ST01/5Fr) is an over-the wire-type 120-cm straight catheter with a 13-cm soft atraumatic tip that enables smooth crossing and deep engagement with a low risk of vessel injury. The smooth inner lumen of 5CG (1.50 mm inner diameter) allows for the consistent passage of a 1.25- or 1.50-mm RA burr, although specific precautions are required as described elsewhere in this article. Additionally, a deeply inserted 6-F or 7-F GC can offer the following potential benefits: 1) compared with the 5CG or the GEC, its wider inner diameter accommodates a larger RA burr; 2) it provides stronger back-up support; and 3) the use of a GC simplifies the procedure by reducing the complexity associated with the 5CG or GEC.

**Table 1:** Advantages and Disadvantages of 3 Types of Catheters for Deep Engagement for RA Burr Delivery

	5F Child GC	GC	GEC
Advantages	Easily achieves deep engagement using specific advancement strategies. Ensures smooth delivery of the RA burr within the catheter, in contrast to the challenges experienced with the GEC.	Easily achieves deep engagement using specific advancement strategies. A larger burr size compared to the 5CG and GEC can be used, depending on the inner lumen size of the GC (6-F GC: ≤1.75-mm burr). Using the GC simplifies the complexities associated with the GEC or 5CG. The RA burr is delivered smoothly within the catheter, unlike the challenges encountered with the GEC.	The rapid exchange design makes it easy to use.
Disadvantages	Available burr size is 1.25 or 1.50 mm.	The risk of coronary ischemia or vessel injury may be higher compared with other catheters.	Advancement of the RA burr through the GEC often fails at the entry port. Specific advancement strategies cannot be used. The available burr size is limited by the inner diameter of each product.

5CG = 5-F child-guide catheter; GC = guide catheter; GEC = guide extension catheter; RA = rotational atherectomy.

Compared with conventional RA for proximal lesions using a 7-F or 8-F GC, the DELIVER technique with a 5CG requires the following precautions. Advancing a 1.5-mm burr through the 5CG may create friction, potentially leading to unintentional removal of the ROTAWIRE. In such cases, it is recommended to advance the burr slowly in DynaGlide mode while carefully maintaining wire position. Even minor kinking of the catheter or wire may increase resistance, leading to burr advancement failure or loss of wire position. Therefore, it is essential to confirm that the catheter and wire are free from kinks along the entire access route, including the coronary artery.

The efficacy of a GEC-facilitated RA via deep insertion of the GEC and burr delivery has been reported recently.^15^^,^^16^ Among 6F GEC, only the GUIDEZILLA is compatible with the 1.25-mm burr; all 7-F GEC are compatible with both the 1.25- and 1.50-mm burrs.^17^ Nevertheless, a significant and unresolved issue with this technique is that the advancement of the RA burr through the GEC frequently fails at the catheter’s entry port (Figure 2C), even when using compatible RA burrs. In such scenarios, the RA burr needs to be preset into the GEC outside the patient’s body; this may enable concomitant advancement of the GEC with the RA burr. However, this approach will be harmful or almost impossible with a high risk of proximal vessel injury, because it requires the direct passage of GEC through the proximal vessel without the aid of 3 specific catheter advancement strategies.

In case 1, GEC-facilitated RA was attempted initially. However, the RA burr was unable to pass through the entry port of the GEC.^17^ Even under these challenging circumstances, the DELIVER technique using a 5CG ensured the successful delivery of the RA burr and subsequent performance of the RA. Once the 5CG or GC has passed through the tortuous vessel or obstacles, the smooth inner lumen of the catheter reduces resistance, enabling consistent passage of the burr. This represents a distinct advantage of the DELIVER technique compared to GEC-facilitated RA (Table 1).

Compared with conventional RA for proximal lesions using a 7-F or 8-F GC, the DELIVER technique requires the following precautions. Although this technique can be performed with various GCs, it is advisable to use soft-tip GCs, such as Heartrail (Terumo) or Hyperion (Asahi), that lack significant curvature (eg, Judkins Right, Judkins Left, Ikari Right, or Ikari Left) to decrease the risk of proximal vessel injury. Given the risk of coronary ischemia or injury during catheter insertion, careful attention must be paid to ensure that the proximal coronary artery has sufficient diameter to accommodate both deep catheter engagement and the subsequent RA. Particular care should also be given to the position and stability of the deeply engaged catheter, mother GC, and ROTAWIRE during ablation, as unintentional removal of a deeply inserted catheter can make reinsertion extremely difficult without employing specific catheter advancement strategies.

The Diamondback 360 Coronary Orbital Atherectomy System has demonstrated effectiveness in managing severe distal calcifications in tortuous vessels, particularly when used in conjunction with a GEC.^18^ Compared with Orbital Atherectomy System, RA offers superior lesion crossability owing to its significantly higher ablation power and the ability to perform forward ablation, enabled by its distally located burr.^19^ Therefore, when the distal target lesion is heavily calcified and resistant to balloon crossing or dilation, the DELIVER technique may offer a more optimal treatment strategy.

## Conclusions

The DELIVER technique is a safe and effective strategy that simplifies RA burr delivery, enabling successful RA of distal calcified lesions in the presence of proximal vessel tortuosity or other obstacles. Furthermore, it protects the proximal vessels from potentially fatal injuries.

## Data Availability Statement

The data that support the findings of this study are available from the corresponding author upon reasonable request.

## Funding Support and Author Disclosures

Drs Kaneko, Kashima, and Kuramitsu have served as proctors for Rotablator for Boston Scientific. All other authors have reported that they have no relationships relevant to the contents of this paper to disclose.

## Appendix

Video 1 Baseline Coronary Angiography

Video 2 Failure to Deliver the 1.25-mm Burr to the Distal Lesion

Video 3 Deep Insertion of a 5-F Child Guide Catheter Beyond the Tortuous Segment Using the Distal Balloon Anchoring Technique

Video 4 Rotational Atherectomy of the Distal Target Lesion, Supported by a 5-F Child Guide Catheter

Video 5 Deep Insertion of the Guide Catheter Beyond the Tortuous Segment, Assisted by Distal Balloon Anchoring

Video 6 Rotational Atherectomy of the Distal Target Lesion, Aided by Deeply Inserted Guide Catheter

Video 7 Deep Advancement of the Guide Catheter Beyond the Ostial Stent

Video 8 Rotational Atherectomy Using a 1.75-mm Burr
